# Tips and Tricks for Validation of Quality Control Analytical Methods in Good Manufacturing Practice Mesenchymal Stromal Cell Production

**DOI:** 10.1155/2018/3038565

**Published:** 2018-09-04

**Authors:** Mariele Viganò, Silvia Budelli, Cristiana Lavazza, Tiziana Montemurro, Elisa Montelatici, Stefania de Cesare, Lorenza Lazzari, Anna Rosa Orlandi, Giovanna Lunghi, Rosaria Giordano

**Affiliations:** ^1^Department of Transfusion Medicine & Hematology, Laboratory of Regenerative Medicine-Cell Factory, Fondazione IRCCS Ca' Granda Ospedale Maggiore Policlinico, Milan, Italy; ^2^Department of Clinical Sciences and Community Health, EPIGET Lab, Università degli Studi di Milano, Milan, Italy; ^3^Clinical Laboratory, Fondazione IRCCS Ca' Granda, Ospedale Maggiore Policlinico, Milan, Italy

## Abstract

Mesenchymal stromal cells (MSC) for cellular therapy in European Union are classified as advanced therapy medicinal products (ATMPs), and their production must fulfill the requirements of Good Manufacturing Practice (GMP) rules. Despite their classification as medicinal products is already well recognized, there is still a lack of information and indications to validate methods and to adapt the noncompendial and compendial methods to these peculiar biological products with intrinsic characteristics that differentiate them from classic synthetic or biologic drugs. In the present paper, we present the results of the validation studies performed in the context of MSC development as ATMPs for clinical experimental use. Specifically, we describe the validation policies followed for sterility testing, endotoxins, adventitious viruses, cell count, and immunophenotyping. Our work demonstrates that it is possible to fully validate analytical methods also for ATMPs and that a risk-based approach can fill the gap between the prescription of the available guidelines shaped on traditional medicinal products and the peculiar characteristics of these novel and extremely promising new drugs.

## 1. Introduction

Manufacturing of pharmaceutical and biopharmaceutical products is subject to standardized quality systems regulated by the Good Manufacturing Practice (GMP) rules [1]. Mesenchymal stromal cells (MSC) represent cell therapy products that under the European Union regulation [2] are classified as advanced therapy medicinal products (ATMPs). Consequently, their production must take place according to GMP standards. The quality control department of a medicinal product manufacturing plant has the aim to guarantee the quality of the product that relies on the evidence of a clear relationship between accurate measurements and critical quality attributes of the product such as safety, identity, purity, and potency. These issues are well described in specific guidelines of European Medicines Agency (EMA) [3]. Safety derives from the demonstration that the product does not contain adventitious agents: bacteria, fungi, and viruses as well as any other components that might represent a hazard for the patient who will receive it; the identity of the cellular components ensures the presence of the active substance and may consist of phenotypic and/or genotypic profile definition; purity demonstrates that the cell therapy product contains at high concentration the active substance and is free from other unwanted cell populations, as far it concerns the desired therapeutic effect. Lastly, potency assay measures the required biological activity in the final cell product, in relationship with the mechanism of action in general or for any defined clinical purpose.

Validation means in this context the successful demonstration of manufacturing and quality consistency, and it is the action of providing that any process, procedure, method, or activity actually and consistently fulfill specific requirements. In particular, according to International Conference on Harmonization Q2 (ICH Q2 R1) Guidelines [4], validation of each analytical method is required with the purpose to demonstrate that the procedures and the test adopted from the quality control laboratory are suitable for the intended use, so they are appropriate to give results in terms of quality attributes, as described above. A validation activity is generally composed of four steps: (1) qualification of personnel and equipment used as prerequisite for all the operations; (2) description of the validation strategy in written and approved validation protocols; (3) performance of the validation experiments; and (4) collection of the results and considerations in a validation report [5]. The validation protocol should clearly define the roles and the responsibilities of each person and element involved in the validation performance, such as equipment, supplies, reagents, reference materials and standards and, above all, the validation parameters and the acceptance criteria that guarantee the fulfillment of the validation specifications. The ICH Q2 (R1) guidelines define the following parameters that should be considered for validation: accuracy, precision (repeatability and intermediate precision), specificity, detection limit, quantitation limit, linearity, and range.

The strategy and the acceptance criteria for the methods to detect microbial contamination in pharmaceutical products (microbiological examination, bacterial endotoxin, and mycoplasma) are described in the European Pharmacopoeia (Ph. Eur.). The aim of their validation is to determine if a specific product contains substances that may interfere with the results of the analysis. Since ATMPs for their nature are not inert products, appropriate considerations and adaptation strategies are required, in regard to their clinical application, to design an accurate validation study.

It is much more challenging for an ATMP quality control department to validate noncompendial analytical methods (those methods that are not included and described in the official Ph. Eur.), especially in terms of identity, purity, and potency. In addition to the limited availability of appropriate standards and reference material, the lack of specific monographs and guidelines makes the validation work even more difficult in this field.

Despite being an important issue for the GMP production of ATMPs, in the literature, there are few papers regarding specific validation strategies [6–8] with very different approaches.

It is important to notice that recently specific GMP guidelines for ATMPs have been published [9], and for the first time, a distinction between investigational ATMPs (at least in the early experimental clinical phases) and authorized ATMPs (products that have reached the marketing authorization) is stated. As concerning the first class of products in this document is clearly declared that full validation of analytical procedures is not required, but demonstration of the methods' suitability may be sufficient, whereas validation is expected for clinical ATMPs in advanced experimental phases. In our experience [10], risk assessment should always drive the ATMP developer choices, and based on this approach, we chose to validate all the methods whose results are used to release investigational ATMPs. We are indeed convinced that only with an accurate, specific, and precise method it is possible to be confident of the results that can support the knowledge of our cellular products and so the way towards its authorization.

So, the aim of this paper is to give a clear explanation of how we designed validation of compendial and noncompendial methods to determine safety (microbiological determination, bacterial endotoxins, and adventitious viruses), identity, and purity (cell count and immunophenotyping) for quality control of GMP MSC, requested as release criteria for early-phases clinical trial.

## 2. Materials and Methods

### 2.1. Prerequirements: Validation of Instruments, Supplies, and Reagents and Personnel Qualification

According to GMP guidelines [1], validation of instruments, supplies, and reagents have been performed as already described [11]. Briefly, the instruments were subjected to installation qualification (IQ), in accordance with the manufacturer specifications and to operational qualification (OQ). Reagents upon receipt were properly checked against specifications and recorded. Authorized GMP staff for quality control department follows a continuous training program. Duties of the personnel involved in quality control procedures and quality control manager's responsibilities were clearly described in written analytical protocols and in job description, as requested by the GMP guidelines.

### 2.2. Validation Plan and Definition of ICH Parameters

For each method, the validation strategy was described in detail in the validation protocol that reported the chosen ICH Q2 (R1) parameters [4], the type of analysis, the number of runs and replicates, the formulas used for calculation, the acceptance criteria, the instruments, the operators involved, and the time schedule for the completion of the validation study. All the results and the analysis were recorded in a report that is approved by the Responsible of Quality Control (RQC) Department. If the validation criteria were not met, the RQC managed this condition as a “noncompliance,” identified and corrected the causes for failure, and rescheduled the validation activities by issuing a new plan.

The following parameters were used in the validation studies herein described.

(i) Specificity: the ability to assess unequivocally the “analyte” in the presence of components which may be expected to be present. Typically these might include impurities.
(ii) Accuracy: the closeness of agreement between the value which is accepted either as a conventional true value or an accepted reference value and the value found. In our noncompendial validation analysis, accuracy can be expressed as follows:
  (1) Accuracy error (*E*_A_) is calculated according to the following formula:
    (1) $$\begin{array}{c} \left(E_{A}\right)=measured\;value-expected\;value. \end{array}$$
  (2) Accuracy (as for percentage values) is calculated according to the following formula:
    (2) $$\begin{array}{c} A=\frac{measured\;value}{expected\;value}. \end{array}$$
(iii) Precision: the closeness of agreement (degree of scatter) between a series of measurements obtained from multiple sampling of the same homogeneous sample under the prescribed conditions. Precision was here considered at two levels: repeatability (intra-assay precision) and intermediate precision (interassay precision), and it was calculated by considering the percentage of the coefficient of variation (CV) between the series of measurements, calculated by the formula:
  (3) $$\begin{array}{c} CV\left(\%\right)=\left(\frac{standard\;deviation}{mean}\right)*100. \end{array}$$
(iv) Detection limit (limit of detection, LoD): the lowest amount of analyte in a sample which can be detected but not necessarily quantitated as an exact value.
(v) Linearity: the ability (within a given range) to obtain test results which are directly proportional to the concentration (amount) of analyte in the sample. We calculated it by considering the correlation coefficient *R* square (*R*^2^) between 1 and 0.9.
(vi) Range: the interval between the upper and lower concentration (amounts) of analytes in the sample (including these concentrations) for which it has been demonstrated that the analytical procedure has a suitable level of precision, accuracy, and linearity.

### 2.3. Reference and Retention Samples for Validation: Cell Source and Manufacturing

According to Annex 13 of the GMP guidelines [12], all the validation methods were performed with reference or retention samples of the final product that were represented by MSC from cord blood (CB) and bone marrow (BM). Briefly, the starting material, CB or BM, after quality control analysis was introduced in our class B-GMP facility and was seeded in alpha modified Eagle medium (Macopharma, Mouvaux, France) supplemented, respectively, with gamma-irradiated foetal bovine serum (FBS) of Australian origin (Gibco, Life Technologies, Carlsbad, CA, USA) or platelet lysate (Institute für Klinische Transfusionsmedizin und Immungenetik Ulm Gemeinnützige GmbH, Ulm German), at the concentration of 50,000 total nucleated cells (TNC)/cm^2^ in culture chamber system (Corning, Lowel, MA).

After 24–72 hours, nonadherent cells were removed by washing with phosphate-buffered saline (PBS) (Macopharma) with complete medium change. The cultures were daily monitored for colony appearance, and the culture medium was changed every three days. At 80% confluence, the cells were detached using 25 mL/layer of TrypLE-Select (Gibco, Life Technologies) and subcultured in the same culture conditions at the concentration of 1000–4000 MSC/cm^2^. At each passage, MSC were washed and cryopreserved with 10% DMSO (CRYOSERV, Mylan Institutionals, Canonsburg, PA, USA), 10% human serum albumin (HSA; Kedrion, Lucca, Italy), in a saline solution (Baxter, Deerfield, IL, USA), in cryo-bags (CryoMACS, Miltenyi Biotec, Bergisch Gladbach, Germany) as retention samples (representative samples of final product in fully packaged unit), and/or in cryovials (Laboindustria, Padova, Italy) as reference samples. Immediately after the addition of the cryopreservation solution, the bags and vials were loaded into a controlled-rate freezer (PLANER Kryo Biorep, Milano, Italy), programmed at −1°C/min until −45°C and then at −5°C/min until −110°C. The frozen units were transferred to vapor-phase liquid nitrogen and stored into dedicated tanks. The freezing curve was validated and recorded in the batch record. For validation protocols, both fresh and cryopreserved MSC were used according to the validation and clinical application.

### 2.4. Standard and Positive Controls

#### 2.4.1. Microbiological Contamination

Microbiological strains (ATCC Manassas, VA, USA) were chosen in accordance with Ph. Eur. 2.6.27 [13, 14], at the version in use at the moment of validation. The lyophilized bacterial strains, yeast, and fungus were appropriately prepared and isolated in Casein Soybean Digest Agar (CASO Agar) and Sabouraud, right-agar (SDA) plates (Merck Millipore, MA, USA). After incubation at the optimum growth conditions, the strains were stored at 4°C ± 2. For use, they were recultivated in agar plates in specific conditions and after measuring the quantity as absorbance (*λ* 625), the cell suspensions were diluted to obtain two concentrations, one of 10–100 CFU/mL and one of 100–1000 CFU/mL. Each batch of culture medium was tested for sterility and fertility before use according to Ph. Eur. 2.6.27 (growth promotion test).

#### 2.4.2. Endotoxins

Preloaded and precalibrated, single-use disposable Endosafe® PTS Cartridges (Charles River Laboratories, Charleston, SC, USA) were used for validation. Each cartridge contained standard endotoxin (CSE) at 0.05 endotoxin unit (EU/mL).

#### 2.4.3. Adventitious Viruses

Positive controls for enterovirus, adenovirus, human cytomegalovirus (CMV), and Epstein-Barr Virus were provided by the Quality Control for Molecular Diagnostics (QCMD) in the context of the International External Quality Assessment (EQA) of our laboratory. The working viral loads were established considering the quantity results shown on the report of the external quality program.

### 2.5. Microbiological Examination Validation

This validation was performed on three batches of CBMSC and on three batches of BMMSC with the purpose to verify if any component of the matrix in which the final product is resuspended has antibacterial activity and may therefore interfere with the results of the test. The matrix solution for CBMSC as a cryopreserved product is made of normal saline, HSA, and DMSO at the concentration described above, while the BMMSC as fresh product was resuspended in HSA and normal saline (5% vol : vol).

The validation scheme and the evaluated parameters are displayed (Table 1). The analysis was carried out for direct inoculation into the microbial culture medium under the class A-GMP laminar flow hood in class B-GMP surround. Continuous particle count monitoring and environmental and operators' microbiological controls were performed [11]. Accordingly to Ph. Eur. [13], the minimum volume to be tested is 1% on the maximum volume of the batch, so for CBMSC was 10 mL (maximum volume of the batch = 1000 mL) and 0.1 mL for BMMSC (maximum volume of the batch = 10 mL). Therefore, in order to have a suitable sample for performing sterility test on the final product, the cell suspension was divided into sterile tubes (10 or 0.1 mL for tube) and to each tube, an inoculum of each microorganism was added at two concentrations, 10–100 CFU/mL and 100–1000 CFU/mL, respectively. In this way, for each microbial strain, two levels of contamination were obtained: 1–10 CFU and 10–100 CFU. The preparations (sample + microorganism) were added to the bottle containing two specific culture media: fluid thyoglicollate medium—FTM (Millipore) for *Staphylococcus aureus*, *Pseudomonas aeruginosa*, *Clostridium sporogenes*, *Propionebacterium acnes*, *Streptococcus pyogenes*,* Micrococcus luteus* and *Yersinia enterocolitica* and soyabean casein digest medium—TSB (Millipore) for *Bacillus subtilis*, *Candida albicans*, and *Aspergillus brasiliensis*. The samples were incubated at 35–37°C for 14 days, and the results were determined by visual observation. Positive controls were carried out by adding only the microorganisms in the absence of product, while negative controls were performed by adding the sample to culture media only.

### 2.6. Bacterial Endotoxin Test Validation

The method chosen for bacterial endotoxin determination was the chromogenic kinetic method (method D, Ph. Eur. 2.6.14) [15] using Portable Test System (Charles River), an automated and portable spectrophotometric microplate reader equipped with a thermostatically controlled incubation chamber. The limulus amebocyte lysate (LAL) and CSE substrate as positive control are placed within prepackaged cartridges that could be directly read by the instrument at the wavelength of 395 nm, which optimizes the detection of the signal generated by the substrate chromogenic LAL. The maximum sensitivity (*λ*) of the method is 0.005 EU/mL. The validation was carried out on CBMSC, as by the study of the product, it represents a “worst case” in terms of endotoxin limit with respect to and the presence of constituents of the matrix in which it is resuspended (as described in Microbiological Examination Validation).

The validation protocol is briefly summarized in Table 1 and consists essentially in three phases:

1. Assurance of the standard curve criteria: as the CSE is contained in the cartridge, this test was performed by the supplier and each batch of cartridges was provided with a certificate of analysis containing the results of the standard curve test. According to Ph. Eur., the standard curve has been performed on each batch of CSE, with three endotoxin concentrations within specified range on three replicates for each concentration. The linearity of the standard curve, given by the absolute value of *R*^2^, must be ≥0.980.
2. Study of the product: *calculation of the endotoxin limit (EL) and the maximum dilution valid (MDV)*: according to Ph. Eur., EL (EU/mL) was calculated by the formula *K*/*M*, where *K* is the threshold pyrogenic dose of endotoxin per kilogram of body mass (for intravenous administration 5.0 EU/kg) and *M* is the maximum recommended dose of the product per kilogram of body mass. MVD value was calculated using the formula: EL × *C*/*λ*, where *C* corresponds to the concentration of test solution. Since we were dealing with a cell suspension and not with a chemical solution, it is impractical to measure the concentration of our product as mass, biological activity, or volume. Therefore, the value of *C* was fixed to 1, thus implying that each volume unit of the test solution corresponds to one unit of product. *Preliminary test for interfering factors*: this step was performed at various dilutions of the product (according to MVD) in order to find the best dilution not activating and/or inhibiting the enzymatic reaction. The cellular product was diluted in water LAL with a minimum volume of 100 *μ*L. The disposable cartridge contains four channels—two channels with CSE and LAL, which serve as the positive control channels, and two channels only with LAL. For each dilution, 25 *μ*L of testing sample was charged in the four wells of a cartridge. The negative control was represented by LAL reagent water (Charles River) in at least two replications. The instrument gave a report in which the following results were indicated: onset time (time necessary to achieve the detectable level of predetermined absorbance); coefficient of variation (CV) of the two replications of the positive controls and samples; and spike recovery that allows to check for any interference (inhibition and stimulation), which can determine an alteration of the endotoxin recovery. In order to have a valid result, the onset time of negative control and no spiked samples must be greater than the onset time of *λ*, the percentage of CV must be ≤10%, and the recovery spike rate directly calculated by the instrument as measured of accuracy must be between 50 and 200%.
3. Test for interfering factors using the chosen dilution on three batches of product to confirm the preliminary test.

### 2.7. Adventitious Viruses Analysis Validation

The validation was designed with the aim to demonstrate the ability of the test to detect, in our biological samples, the viruses that could be accidently introduced by the operators during processing of cultures. In particular, to disclose viruses belonging to the herpes viruses family i.e., cytomegalovirus (CMV) and Epstein-Barr virus (EBV), CMV ELITe MGB® Kit and EBV ELITe MGB Kit (both ELITechGroup, Torino, Italy) were used. They represented quantitative real-time methods for diagnostic use in DNA extracted from whole blood samples, plasma, and cerebrospinal fluid (CSF). To detect simultaneously the presence of fifteen respiratory viruses (Table 2), Seeplex RV15 OneStep ACE Detection (Seegene, Seoul, Korea), a qualitative approach based on a real-time one-step RT-PCR assay that is designed to detect respiratory viruses in nasopharyngeal aspirate, nasopharyngeal swab, and bronchoalveolar lavage, was chosen. As summarized in Table 1, the first phase of the validation was aimed at defining the number of cells needed for optimal DNA/RNA extraction and amplification, also considering possible inhibiting effects from DMSO and genomic DNA that are obviously present in the samples. Three batches of reference cryopreserved CBMSC were thawed, counted, and prepared in three different cell doses (0.1, 0.5, and 1 × 10^6^ cells). The pellet was resuspended in lysis buffer ATL (Qiagen, Hilden, Germany), and DNA and RNA were extracted using an automatic extractor EZ1 (Qiagen) in a final volume of 120 *μ*L. For MGB Kit, PCR mix was added 1 : 1 in volume to genomic extract and the negative control of extraction. For each sample, two amplification reactions are carried out in a thermocycler (7300 Real-Time PCR System, Applied Biosystems, Foster City, CA, USA): the first one was aimed to detect the MIEA gene (exon 4), for CMV, or the gene encoding the EBNA1 protein, for EBV and the second one was directed against the human beta globin gene, to verify that extraction and amplification were successfully carried out without inhibitions. For viral title determination, a standard curve was set using four scalar concentrations of plasmid DNA (1 × 10^2^, 1 × 10^3^, 1 × 10^4^, and 1 × 10^5^ copies) of the two viruses, according to kit's instructions. Only a standard curve with *R*^2^ > 0.9 was accepted.

For respiratory viruses, amplification was done adding 8 *μ*L of extract to 17 *μ*L of different master mix according to the manufacturer's instructions. The kit contains two different controls: the first one is the PCR control (PCRC) that allows to verify if the amplification has been carried out correctly without inhibition by substances contained in the samples and the second one is the whole process control (WPC) which is a target against the Rnase that allows to verify if the whole process, from the extraction to the amplification, has happened correctly without inhibition. In order to assess the specificity test on MSC, the cells (BMMSC and CBMSC) were inoculated with two viral loads of known positive standard, according to the results of external quality program's report. Since it was not possible to use all the fifteen respiratory viruses to be inoculated to the cells, we decided to use as positive standards a representative DNA virus (adenovirus) and a RNA virus (enterovirus). The viral quantities used to spike the cells were comparable of a low and high viral load in a positive patient sample: 359 and 1436 copies for CMV, 656 and 1313 copies for EBV, 362 and 723 copies for adenovirus (AdV), and 1500 and 3000 copies for enterovirus (HEV).

Samples spiked with HEV were performed with or without proteinase K treatment, to exclude inhibition of the RNA extraction due to treatment at 56°C (needed for proteinase K).

To assess sensibility, accuracy, and precision, three different batches of CBMSC were charged with a viral load tenfold over the sensitivity cutoff declared by the manufacturer, 200 and 1000 copies for AdV and HEV, respectively, and 85 and 95 copies for CMV and EBV, respectively. For the recovery calculation of the genome equivalent (gEq) copies in the sample, it was not possible to apply the formula indicated by the datasheet (built for plasma samples), so results were expressed as quantity of CMV and EBV target DNA (gEqu/reaction) that was present in the sample reaction and in positive control (consisting of viral nucleic acid alone). The quantity parameter was calculated by comparing Ct values of each sample and the standard curve. For respiratory viruses, being the kit qualitative, it was not possible to demonstrate a quantitative recovery. Two negative controls were performed: negative control of extraction, by processing water under the extraction conditions, and negative control for amplification, by putting water in the mix for amplification. Precision was assessed within technical replicates and within the three batches of CBMSC.

### 2.8. Cell Count Validation

**Figure 1:**
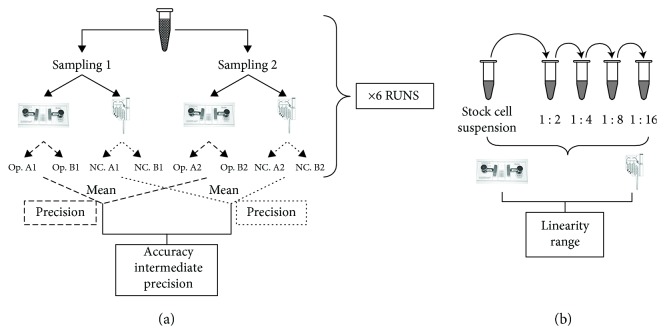
Strategy design for cell count validation. (a) Two samplings of cell suspension (*n* = 6) were counted each by two qualified operators (Op.) in hemocytometer (Burker chamber) for the manual method and by two cartridges for the automated method (Nucleocassette-NC.). The mean of all the values was used to calculated the accuracy (as accuracy error between the manual and the automated total and viable cell count) and the intermediate precision (interassay coefficient of variation, CV). The intra-assay CV was calculated considering the values of each cell suspension count for each method. (b) The cell suspension was then serially diluted and counted for comparing linearity of the three methods and the optimal range of cell concentration to count.

The protocol was designed to validate an automated method for MSC counting by “NucleoCounter®” system (ChemoMetec, Allerod, Denmark) in terms of accuracy, precision, and linearity in comparison to the manual cell count method by the hemocytometer (Burker chamber). Nucleocounter is a portable device based on integrated fluorescence microscope principle that allows to count total and viable cells stained with the propidium iodide (PI), immobilized inside the charger NucleoCassette. Reference samples of MSC (three batches of CBMSC and three batches of BMMSC) were resuspended in a volume between 1 and 20 mL of PBS, in order to test different concentrations of cells. As shown in Figure 1 for each cell suspension, two different samplings were counted in duplicate for total and dead cell. For the first, the cells were pretreated with a buffer of lysis (ChemoMetec), in order to allow the PI to stain all the cell suspension. The cell stock solution was then serially diluted (1 : 2–1 : 4–1 : 8–1 : 16) and counted with the two methods.

The count by Burker chamber was performed by two qualified operators: 10 *μ*L of cell suspension was loaded and five fields were counted.

The following parameters and acceptance criteria were set up: (1) accuracy: calculated as accuracy error between the hemocytometer count and the measured value (nucleocounter total and vital count) and the acceptance criteria was fixed as −5 ≤ E_A_ E ≤ +5; (2) linearity: for each method in the diluted serial counts calculated as *R*^2^ between 0.9 and 1 and (3) precision: estimated for repeatability (intra-assay) and intermediate precision (interassay) with a CV less than 20%.

### 2.9. Immunophenotyping Validation

**Figure 2:**
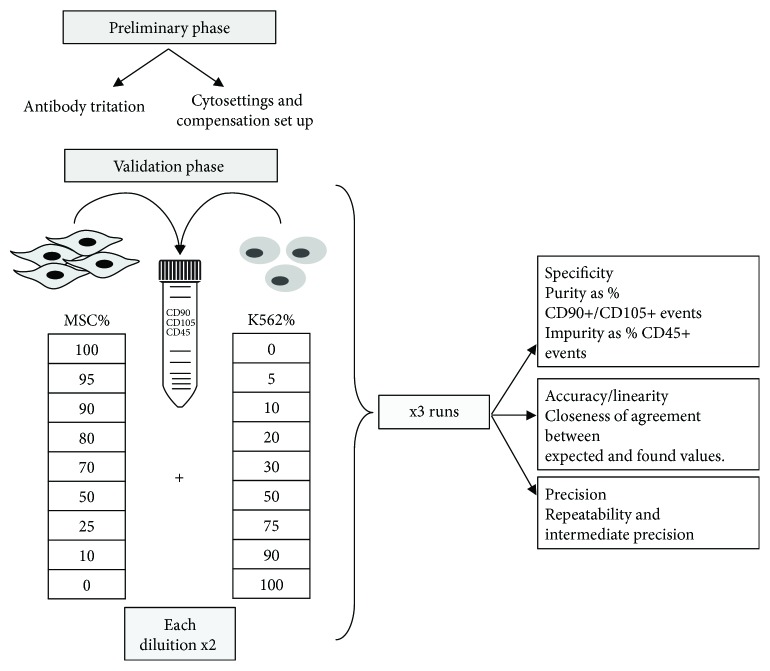
Strategy design for immunophenotyping validation. The preliminary phase of the validation study for MSC immunophenotyping analysis consisted in the antibody titration and the settings of the instrument for the intended use. MSC (*n* = 3) were mixed in duplicate with different concentrations of CD45-positive cells (K562), stained for CD90, CD105, and CD45 and acquired by flow cytometry. Specificy, accuracy, linearity, and precision were determined.

The validation design for the immunophenotyping analysis (Figure 2) was preceded by a preliminary phase consisting of, at first, the titration of each antibody used and then the instrument settings (including fluorescence voltages and compensation setup). The validation phase consisted in the evaluation of the parameters in compliance with ICH Q2. We used the cytometer FACS CANTO II (Becton Dickinson, BD Bioscience, San Jose, CA, USA), whose reproducibly setup was checked with CS&T beads (BD) before each acquisition, according to manufacturer's instructions.

As reference samples, three batches of MSC (*n* = 2 CBMSC, *n* = 1 BMMSC) and K562 cells were used as positive and negative standard (stMSC and stK562), respectively. K562 is a myeloid cell line positive for hematopoietic marker CD45 and with dimension analogous to those of MSC. Due to this feature, acquisition settings can remain unchanged as compared to MSC alone, thus allowing the simultaneous visualization of both cell types that can be discriminated based on the expression of CD45.

For the antibody titration, 1 × 10^5^ MSC per 100 *μ*L were stained for 20 minutes at room temperature in the dark with the following antibodies at different concentrations (0, 1, 2.5, and 5 *μ*L): CD90 PE-Cy7 (BD), CD105 PerCP-Cy 5.5 (BD), and CD75 APC (BD). After incubation, the cells were washed with PBS, and analyzed with the DIVA software program (BD). A minimum of 10,000 events were analyzed. The samples were evaluated in terms of percentage of positive cells and mean fluorescence intensity ratio (MFI-R), calculated as the ratio of the MFI of the positive cell population on the MFI of the negative cell population (unstained cells). These values were plotted as a function of antibody quantity to determine which dilution is the best for each antibody to be used in the following step of validation. The optimal titer was identified as the lower quantity allowing the greatest discrimination between positive and negative cells, that is, the first concentration allowing the reaching of the plateau.

The instrument was set up for the acquisition protocol by manual calibration that was performed by preparing a working standard solution (WstS), consisting of 50,000 MSC (stMSC) mixed to 50,000 K562 cells (stK662). Seven tubes were prepared as follows: unstained WstS, WstS stained with anti-CD90 FITC (BD), WstS stained with anti-CD105 PE (BD), WstS stained with anti-CD90 PE-Cy7, WstS stained with anti-CD105 PerCP-Cy 5.5, WstS stained with anti-CD73 APC, and WstS stained with anti-CD45 APC-H7 (BD). The voltages and the compensation settings were verified with stMSC+stK562 stained with the combination of the chosen antibodies mixed together (CD90 PE-Cy7, CD105 PerCP-Cy 5.5, CD75 APC, and CD45 APC-H7). In this analysis, adjustments were made to ensure that no false staining occurs in the dual-color quadrant for any individual fluorochrome. After setting color compensation, the analysis protocol was defined with precise histograms and gating scheme. The samples for the validation step were analyzed in this protocol with no further adjustments.

For the validation phase, positive standard of stMSC (*n* = 3) was combined with negative standard stK562 in 9 different ratios, each in duplicate, according to Figure 2. Each preparation (*n* = 18 total tubes) was stained for CD90, CD105, CD73, and CD45 as described above. The method parameters evaluated were (a) specificity: defined as both purity, that is, the ability to detect the positive markers in MSC, that in our condition is defined by the percentage of CD90+ CD105+ events, and impurity, that is, the ability to detect the negative marker CD45; (b) accuracy: calculated as the degree of agreement between expected and measured percentage results (−5 ≤ accuracy ≤ + 5); (c) linearity for each RUN in the diluted serial analysis: calculated as *R*^2^ between 0.9 and 1 for both purity and impurity; (d) and precision: estimated for repeatability (intra-RUN) and intermediate precision (inter-RUN) with CV less than 20%.

## 3. Results and Discussion

### 3.1. Microbiological Examination Validation

Sterility has always been one of the major and most critical test for ATMP release. In particular, the time to complete the analysis may be an issue for ATMP product with a short shelf-life. In this regard, the paragraph 2.6.27 included in the previous edition of Ph. Eur. might have been incompatible with the need to release this kind of short-living ATMP product, thus making this issue suitable for being considered as a parametric test [16]. As we will more extensively comment below, the same paragraph 2.6.27 in the more recent edition of Ph. Eur. aims to facilitate the use of sterility analytical methods by including alternative approaches and reducing the incubation period, but time still remains an issue for those products that must be released immediately after the completion of the manufacturing process (fresh products). In our experience, some products, such as CBMSC, are cryopreserved before use and so the result of sterility is always available before release. Other products, such as BMMSC, must be released as fresh products and requires alternative approaches for validation and testing. For CBMSC as well as other cryopreserved products, the validation study was designed with the aim to verify if any of the cryopreservation solution components (DMSO, normal saline, and human albumin) could interfere with the detection of microorganisms. The method selected was the direct inoculation of the sample into test media as described in Ph. Eur. Chapter 2.6.27 that fits specifically with cell products while the membrane filtration method described in Ph. Eur. Chapter 2.6.1. may present difficulties when applied to cells. The main challenge of this validation was to define the most representative sample of the final product, in terms of volume and conditions (fresh versus cryopreserved). Regarding the volume to be tested, we decided to assimilate our product to hematopoietic cell preparations for which the Ph. Eur. prescribes to inoculate 1% of the total volume for a final product volume greater than 10 mL. Considering that the number of cells/volume of each CBMSC batch will vary in our manufacturing process (from 300 to 1000 mL), we decided to fix the volume as the maximum one that can be obtained in a standard manufacturing process (1000 mL). For that reason, for validation purposes, we tested 10 mL of final product for each microorganism strain. Regarding the condition of the final product, since CBMSC are cryopreserved and must be thawed before clinical use, sterility testing was validated on a thawed retention sample contained in a cryopreservation bag as the final product. For routine quality controls, the retention sample must be thawed and tested for sterility within three weeks from the completion of manufacturing process and also in the validation study, the same time schedule was followed.

For BMMSC that are released as fresh product, the tested sample for validation was composed by pure BMMSC in a solution made of normal saline and human albumin. The volume chosen for the inoculation with each microbial strain was 0.1 mL, 1% of the total volume that is 10 mL.

For both CBMSC and BMMSC, the results obtained in the validation studies met the preestablished acceptability criteria and specifically (i) the growth of the inoculated microorganisms was observed in the presence and in the absence of the cell product (positive controls) for all the three validation runs, thus indicating that the product does not possess intrinsic antibacterial activity; (ii) no microbiological growth was observed in the negative controls both in the presence of the product and with culture media only (specificity); (iii) the limit of detection was 1–10 CFU, as requested, with and without the product. In particular, it was possible to detect the microorganism with the lowest quantity inoculation that was 2 CFU and the growth of microorganisms was observed by seven days of incubation; and (iv) the samples analyzed by two different operators on two different days had the same growth (intermediate precision).

The recent revised Ph. Eur. Chapter 2.6.27 entitled “Microbiological Examination of Cell-Based Preparations” [14] takes into account the characteristics and the limitation of these preparations, as their shelf-life, that if not cryopreserved, ranges from hours to few days [17], as well as sample composition (in some cases, the cell-based preparation itself can inactivate contaminating microorganisms resulting in a false negative) and/or the sample size (the total volume of a batch could be less than 50 mL, thus limitating the sample size). In this regard, the main changes to previous version concern a greater flexibility for the incubation temperature(s), a change in the list of microorganisms to be tested (*Yersinia enterocolitica* is replaced by *Micrococcus luteus* that is more appropriate as it is a common contaminant of cell-based preparations), and information about the sensitivity to be achieved during validation has also been included. Several authors have already been demonstrated that, for example, automated sterility testing is capable of rapidly detecting low-level contamination, with an average of 2.5 days [18] and within 48 hours [19] for different biopharmaceutical and transplantation products (e.g., pancreatic islets). We are also validating a rapid sterility testing with the aim to demonstrate that it is accurate, sensitive, and specific (results not shown).

### 3.2. Bacterial Endotoxin Test Validation

LAL evaluation is the most sensitive and specific test currently available to detect and measure bacterial endotoxins, defined as “pyrogens” as they induce fever and other adverse reactions caused by inflammatory mediators.

In Ph. Eur. [15], six different methods to determine endotoxins in pharmaceutical products are described. In this paper, we present the results of the validation using a kinetic chromogenic test in which the reaction time of the sample is compared with that of control standard endotoxins (CSE). The reaction time of the sample is defined “onset time” (time to achieve a given level of optical density). The first aim of the validation design was to verify the suitability of the reagents and specifically the sensitivity of the CSE. For the chromogenic method, this step is represented by the assurance of the standard curve criteria. We verified that the three values of endotoxin tested in the cartridge (0.5–0.05–0.005 EU/mL) and reported in the standard endotoxin certificate of analysis include a central value that corresponded to the amount of endotoxin loaded as positive control in the cartridge itself and that the last value corresponded to sensitivity (*λ*). Moreover, we checked that the standard curve provided by the supplier had a linearity value of 0.998. Secondly, the validation on the product must be performed with several purposes: (1) to identify possible interference by the product itself, (2) to show that the chosen dilution does not interfere, and (3) to eliminate the possible sources of interference by different means (e.g., heating). Before starting with the validation study, the specific endotoxin limit (EL) and the maximum valid dilution (MVD) must be calculated for each specific product as described in Materials and Methods. The calculation of EL and MVD for LAL test on ATMP product is a good example of how difficult is the adaptation of compendial methods that have been set on standard pharmacologic products. In fact, Ph. Eur. states that for new medicinal product, EL and MVD must be calculated by the user basing on administration routes and other pharmacological parameters, but there are still no defined guidelines that explain the rational to be followed to define these values for ATMP. Moreover, in the literature, some authors just indicate the values without giving any justification on the calculation made to determine them [6], while others chose the EL value of 0.5 EU/mL [7], as requested by the Food and Drug Administration for general medical devices or for individual drug products that has a maximum human dose of 10 mL/kg [20]. In our condition, we tried to respect as much as possible the “spirit” of Ph. Eur. criteria by calculating the EL and MVD of our products in consideration of their clinical use. Specifically, the EL and MVD in our conditions were calculated as follows:

(4) $$\begin{array}{c} EL=\frac{K}{M}. \end{array}$$

where *K* is 5 EU/kg (as requested by Ph. Eur. for parenteral administration) and *M* is the maximum cellular dose infused per kilogram in a single-hour period that in our condition is the maximum volume of infusion per kilogram. For CBMSC, the maximum volume infused considering a standard adult body weight of 70 kg in 1-hour period is 80 mL, so

(5) $$\begin{array}{c} M=\frac{80\;mL}{70\;kg}=1.14\;mL/kg. \end{array}$$

Therefore, EL for CBMSC is

(6) $$\begin{array}{c} EL=\frac{5\;EU/kg}{1.14\;mL/kg}=4.39\;EU/mL. \end{array}$$

For BMMSC, the maximum volume infused considering a standard adult body weight of 70 kg in 1-hour period is 10 mL, so

(7) $$\begin{array}{c} M=\frac{10\;mL}{70\;kg}=0.14\;mL/kg. \end{array}$$

Therefore, EL for BMMSC is

(8) $$\begin{array}{c} EL=\frac{5\;EU/kg}{0.14\;mL/kg}=35.7\;EU/mL. \end{array}$$

Considering both clinical protocols, here, we reported the validation of LAL on CBMSC that represents the “worst case” as it has the lowest EL and the most complex cell matrix (including DMSO).

So we calculated MVD:

(9) $$\begin{array}{c} MVD=EL/\lambda =\frac{4.39\;EU/mL}{0.005\;EU/mL}=878. \end{array}$$

For preliminary test on the interfering factors, the product was tested at different dilutions, in order to identify the most suitable noninterfering dilution. The chosen sample dilutions were 1 : 30; 1 : 90; and 1 : 180.

The results obtained (Table 3) were satisfied for all the three dilutions of the product. We can conclude that the product was not interfering and the LAL test was valid. It was decided to use the lowest dilution of 1 : 30 in the next phase.

In order to confirm that the chosen dilution did not have any interference, the test was repeated by three different operators on three batches of product, and for all the experiment session, the acceptance criteria were met. Finally, we calculated specific sensitivity of the test (PSS) as follows:

(10) $$\begin{array}{c} \lambda \times chosen\;dilution=0.005\;EU/mL\times 30=0.15\;EU/mL. \end{array}$$

So we successfully performed LAL validation in our cell product using a sensitive and rapid quantitative method, with the encouraging result that the sensitivity of our test is much higher than that of the EL to be confident in the detection of the endotoxins. The specification for endotoxin test for the product release was chosen according to the EL and the sensitivity of the test.

Regarding the decision to remove interfering factors by treating the product, we decided not to make any manipulation in order to test the most representative sample of the released final product with an acceptable final dilution. Our results were consistently supporting this choice, but it is indeed possible in the case of sample interference, to adopt alternative approaches, that may consist, for example, in heating the sample or modifying the pH of the media. In this case, it may be possible to test also a lower dilution.

New frontiers in the application of LAL to ATMPs are represented by the development of alternative methods for complex tissue-engineered products or combined products (cells in combination with biomaterials). In this field, alternative methods have recently been proposed such as cell-based assays that are able to detect material-bound microbial contaminations not detectable with LAL test [21] or immunofluorescent staining assays to evaluate the endotoxin-induced expression of E-selectin. The latter method could give information also regarding the localization of bacterial contamination sources in all steps of the manufacturing process of tissue-engineered product for human use [22].

### 3.3. Adventitious Viruses Analysis Validation

ATMP production carries the risk of adventitious virus contamination, such as airborne respiratory viruses, that can be introduced by the operators during processing. Testing these viruses therefore is an essential quality control step in manufacturing biological medicines. The compendial guidelines Q5 ICH [23] concern products derived from in vitro cell culture, such as interferons, monoclonal antibodies, and recombinant DNA-derived products, including recombinant subunit vaccine that characterized cell bank production. Gombold et al. [24] underline the limit of Q5 ICH (in vivo and in vitro assays) in the detection of viruses in vaccine, as they were developed more than 50 years ago and suggest the need of new detection methods. While regulatory monograph and the literature cover the adventitious virus assessment in vaccine, no specific guidelines are available to our knowledge for the detection of these viruses in ATMPs. Most of the commercial kits, moreover, are validated for specific diagnostic use, in biological material such as blood, nasopharyngeal aspirate, and bronchoalveolar lavage. In our conditions, we validated three of these diagnostic kit, in particular, a quantitative method for citomegalovirus (CMV) and Epstein-Barr virus (EBV) and a qualitative test for respiratory viruses in our cell products.

In order to evaluate the best conditions for extraction and amplification, in a preliminary phase, we tested three thawed batches of CBMSC at different cell doses (0.1, 0.5, and 1 × 10^6^ cells). The results obtained from the internal positive controls demonstrated that also for the lowest amount of cells, both the extraction and the amplification procedures were successful and there was no inhibition by samples' components.

**Figure 3:**
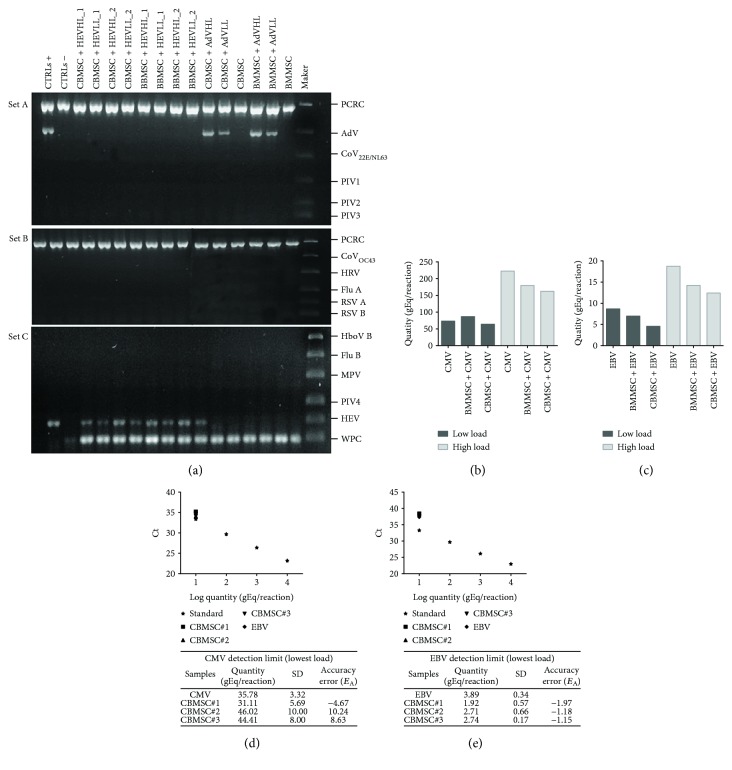
Adventitious viruses validation results. Specificity of the method was assessed spiking MSC (*n* = 2) with a high and low viral loads (HL and LL) of adenovirus (AdV), enterovirus (HEV), cytomegalorovirus (CMV), and Epstein-Barr virus (EBV) (a–c). (a) Results obtained from the qualitative analysis of fifteen respiratory viruses. The samples spiked with HEV were treated (_1) or not (_2) with protease K. Positive controls (CTRLs +) were represented by virus alone (in set A AdV and in set C HEV). A PCR internal control (PCRC) and a whole process control (WPC) were visible in set A and set C. (b–c) Real-time PCR analysis expressed as quantity (gEq/reaction) of CMV and EBV in all the spiked MSC and in the positive controls (CMV and EBV). (d, e) To assess detection limit and accuracy, CBMSC (*n* = 3) were spiked with a lowest viral load of CMV and EBV. Ct of different quantities of DNA plasmid (standard) was plotted with Ct of positive controls and spiked MSC.

In order to assess the specificity of the tests, 0.1 × 10^6^ BMMSC and the same dose of CBMSC were inoculated with a low and high viral load of CMV, EBV, enterovirus (HEV), and adenovirus (AdV). As shown in Figure 3(a), for the respiratory viruses, in all the samples, the band of the internal control (PCRC) and that of the whole process control (WPC) were present; there was a specific signal for AdV and HEV, not only in its positive control but also in the samples spiked with both viral loads and as expected, no specific bands resulted in negative controls. Moreover, we demonstrated that the treatment at 56°C with the proteinase K does not inhibit the RNA extraction.

Also with CMV and EBV, the results provided by real-time PCR reported in Figures 3(b) and 3(c) showed that there is specificity in all the samples spiked with the two low and high viral loads. The results obtained for respiratory viruses, CMV, and EBV were also consistent with the sensitivity declared by the manufacturer. Spiking the cells with a virus quantity near the cutoff of the sensitivity of each kit, we could detect the presence of AdV and HEV in all the samples (data not shown). In particular, for CMV and EBV, the Ct of the spiked samples were correlated to the Ct of the standard represented by plasmid DNA and the positive controls, viruses in the absence of the cells (Figures 3(d) and 3(e)). It was also possible to calculate the accuracy error (*E*_a_) by comparing the virus “quantity” for each batch of CBMSC and of the CMV-and EBV-positive controls, and the acceptance criteria (20 ≤ accuracy error ≤ +20) was satisfied.

Moreover, precision criteria were met, since the coefficients of variation within technical replicates (intra-assay) and between three different batches of CBMSC were ≤ 20%, for both CMV and EBV test.

### 3.4. Cell Count Validation

In order to guarantee the correct cell dose required for different clinical trials, cell count must be accurate. Manual counting with a hemocytometer is the most commonly used method for cell count and is the reference method described in Ph. Eur. (Chapter 2.6.29) [25]. Nevertheless, different automated methods are available on the market that allow to obtain operator-independent cell counting that in a GMP settings becomes extremely relevant to rely on a univocal accurate test. Gunetti et al. [26] explain in detail the validation of a disposable device in comparison with the Burker chamber, while Radrizzani et al [5] performed validation of a flow cytometry method.

In our laboratory, we decided to validate an automated method that allows the count of global and viable total nucleated cells (TNC) in comparison to Burker chamber in terms of accuracy, linearity, and precision (repeatability and intermediate precision).

**Figure 4:**
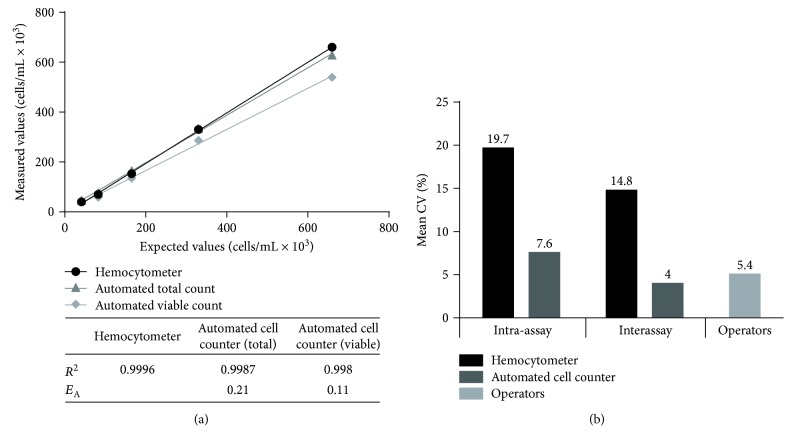
Cell count validation results. (a) Representation of linearity for serial dilution counts by hemocytometer, automated total, and viable cell count. In the table, the linearity (*R*^2^) and accuracy error (*E*_A_) values (considering the hemocytometer count as the expected one) were reported. (b) Intra-assay and interassay precision expressed as CV for all the three counting methods.

The accuracy calculated as accuracy error (difference between the mean of values from two operators by Burker chamber and mean of values of single nucleocassette by Nucleocounter) was within the range of the acceptance criteria −5/+5 (0.07/0.36 as minimum and maximum *E*_A_ for automated total cell count and −0.02/0.29 for automated viable cell count). In particular, we obtained the lowest discrepancy between the manual method and the automated method for viable cell count: mean accuracy error for viable count 0.11 versus 0.21 for total cell count (Figure 4(a) in table). These results demonstrate that the operators are well trained in performing the manual method according to our written SOP and are able to distinguish the cells that are visually intact from those that show “signs of death” as damaged cell membrane.

The dilution experiments (Figure 4(a)) indicate that both the automated and the manual methods maintain a good linearity even at low cell concentrations. The manual method indeed appears to be less precise than the automated one as shown by CV in terms of repeatability and intermediate precision. Notably, the high CV found with the manual method does not appear to be dependent on operator variability; in fact, the CV interoperators are relatively low (Figure 4(b)).

These data demonstrated that the tested automated method is accurate and precise and ensures the linearity of the results obtained to a range of cell dilutions. These results allow also to define the optimal cell concentration range for cell count between 0.2 and 0.75 × 10^6^ cells/mL.

### 3.5. Immunophenotyping Validation

MSC immunophenotypic characterization is fundamental for the identification of the cell product before clinical application. The lack of specific and distinct cell surface markers and the heterogeneity of the characterization studies led, more than ten years ago, the International Society for Cellular Therapy (ISCT) to publish the minimal criteria for defining MSC [27]. In addition to plastic adherence and in vitro differentiation potential, it was defined that MSC are characterized by the expression of CD105, CD73, and CD90 and lack of expression of hematopoietic and endothelial surface markers such as CD14, CD45, CD34, CD11b, HLA-DR, and CD31. Currently, these criteria are still used as accepted standards to define MSC for clinical application. Flow cytometry represents the most widely used method for immunophenotypic analysis also in GMP settings, as it allows a fast, multiparametric analysis of a cell suspension. Nevertheless, as some authors have already underlined [28, 29], assessment of the analytical measurement as sensitivity and linearity for the validation of this method is affected by the lack of cellular reference materials and the difficulty in obtaining adequate controls (e.g., cell lines with varying levels of a given marker expression).

As an important prerequisite of our validation process, we first defined and applied written procedures to set up the instrument that included the titrations of the antibodies for MSC staining and the creation of a specific acquisition protocol with fixed fluorescence settings and compensation (data not shown). In order to assess our ability to detect positive markers (to determine the purity of MSC) and hematopoietic markers (to detect impurity), we chose to spike MSC with different concentrations of a hematopoietic line (K562) that has similar dimension/scatter to MSC. To simplify the analysis, we decided to take into consideration the two positive markers for purity (CD90 and CD105) and a negative marker (CD45) for impurity.

**Figure 5:**
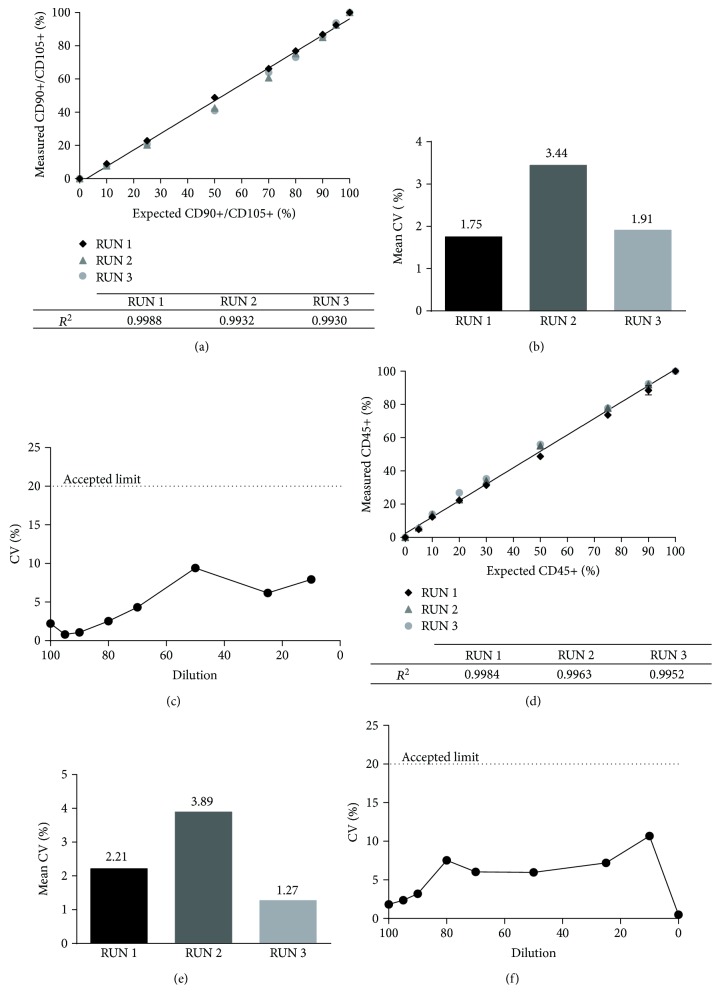
Immunophenotyping validation results. Linearity (a, d), intra-assay (b, e), and interassay (c, f) evaluation for expression of purity (percentage of CD90/CD105+ positive cells) in (a–c) graphs and impurity (percentage of CD45+ positive cells) in (d–f) images.

The results shown in Figure 5 demonstrated that, for both purity and impurity, we obtained a good linearity in all the experiment sets (*R*^2^ > 0.9, Figures 5(a) and 5(d)) and the accuracy value is between −5 and +5. The calculated CV is less than 5% for repeatability (Figures 5(b) and 5(e)) and less than 10% for intermediate precision (Figures 5(c) and 5(d)), so all the acceptance criteria were met. This validation allowed us to have a standardized protocol for MSC purity with the certainty to be able to detect less than 5% of impurities.

## 4. Conclusions

Quality controls of ATMPs are a much jeopardized issue: there are few paragraphs of the pharmacopoeia dedicated to cellular products (e.g., the microbiological controls) while ATMPs are generally poorly represented in the official documents. That is why one of the most demanding and challenging operating field of the persons involved in quality control is to adapt compendial method to the ATMP setting or to validate noncompendial methods.

In the first year of activity of our hospital-based GMP facility, the first approved in Italy in 2007, it was a significant breakthrough to find the way towards efficient and rational validation approaches for the ongoing and future clinical applications. Here, we summarized the most critical validation methods to define cellular safety, identity, and purity in the early phases of clinical trial in order to give a useful tool for other GMP manufacturing centers.

According to the specific GMP guidelines for ATMPs [9], potency assays are expected to be validated prior to pivotal clinical trials. Potency regards the relevant biologic cellular function, and it could be influenced by many variables as the donor variability and cellular population heterogeneity as immunogenicity, senescence, and resistance to cryopreservation that may affect their effectiveness *in vivo* [30]. Moreover, this variability, together with the uncertain mechanism of action and the lack of reference standards, makes the validation strategy difficult to develop. Some groups have already addressed this important issue [31, 32], and their works are very precious to open an “*arena*” of discussion in order to improve the quality profile of ATMPs, thus fostering their reliability as effective and innovative therapeutic tools.

## Figures and Tables

**Table 1:** Validation strategy for “safety” detection methods.

Test method	Ref. Ph.Eur./ICH	Validation steps	Evaluated parameters	Acceptance criteria
Microbiological examination	Ph. Eur. 2.6.27/ICHQ2	Analysis on CBMSC (*n* = 3) and BMMSC (*n* = 3) spiked with 1–10 CFU and 1–100 CFU of each microorganisms Volume of inoculum: 1% of the total batch volume Cryopreserved (CBMSC) and fresh (BMMSC) sample validation	Accuracy	Microorganism growth of medium alone comparable in the presence of the product as confirmation of antibacterial activity of the product
			Specificity	
			Detection limit	
				No growth in the negative controls (specificity)
				Limit of detection: 1–10 CFU
			Precision	
				Same results for each validation run performed by at least two different operators

Bacterial endotoxin test	Ph. Eur. 2.6.14 (method D)/ICHQ2	Assurance of standard curve criteria	Linearity	*R* ^2^ of standard curve ≥ 0.980
		Study of the product (according to clinical use)	EL and MVD calculation	NO-interfering dilution < MVD
		Test of interfering factors	Accuracy (%)	50% < spike recovery < 200%
			Specificity	Onset time of negative control and sample(s) no spike > onset time *λ*
			Precision	CV (intra-assay) ≤ 10%
			Sensitivity	*λ* × chosen dilution

Adventitious viruses analysis	ICHQ3/ICHQ5A(R1)	Preliminary phase	Cell number, extraction, and amplification conditions setup	No inhibition on extraction and amplification for respiratory, CMV, and EBV viruses, with the kit usually used for standard biological diagnostic samples
		MSC spiked with two viral loads of adenovirus, enterovirus, CMV, and EBV	Specificity	Detection unequivocally of the specific viruses in BMMSC and CBMSC which may be expected to be present
		MSC (*n* = 3) spiked with a viral load ten times the cutoff of sensitivity declared by the manufacturer	Accuracy	For CMV/EBV (quantity analysis): quantity of target DNA present in the sample reaction (gEqu/reaction) in spiked MSC samples similar to positive control (−20 ≤ accuracy error ≤ +20)
			Detection limit	Ability to detect the viruses in spiked MSC near the cutoff of the kit
			Precision	CV (intra-assay and interassay) ≤ 20%

**Table 2:** Respiratory viruses detected by the qualitative validated method.

Set A	Set B	Set C
PCR control (PCRC)	PCR control (PCRC)	Bocavirus 1/2/3/4 (HboV)
Adenovirus A/B/C/D/E (AdV)	Coronavirus OC43 (CoV OC43)	Influenza B virus (Flu B)
Coronavirus 229E/NL63 (CoV 229E/NL63)	Rhinovirus A/B/C (HRV)	Metapneumovirus (MPV)
Parainfluenza virus 1 (PIV1)	Influenza A virus (Flu A)	Parainfluenza virus 4 (PIV4)
Parainfluenza virus 2 (PIV2)	Respiratory syncytial virus A (RSV A)	Enterovirus (HEV)
Parainfluenza virus 3 (IV3)	Respiratory syncytial virus B (RSV B)	Whole process control (WPC)

**Table 3:** Preliminary endotoxin test on the interfering factors on the final product at different dilutions.

Parameters	Product dilution			Negative control (LRW)	Acceptance criteria
	1_30	1_90	1_180		
Onset time sample no spike	>1039	>1039	>1039	>1039	>1039
CV between sample replicates (%)	0	0	0	0	<10%
CV between spike replicates (%)	1.3	5.6	2.3	0	<10%
Spike recovery (%)	115	122	114	np	50%–200%
Sample value (EU/mL)	<0.15	<0.45	<0.9	np	<EL

## Data Availability

The data generated or analysed during this study are provided in Results and Discussion.
